# Metabolic Dysfunction-Associated Steatotic Liver Disease (MASLD): From Steatosis to Systemic Metabolic Failure: Classical Pathophysiology, Emerging Systemic Mechanisms and Modifiable Lifestyle Determinants

**DOI:** 10.3390/nu18142316

**Published:** 2026-07-15

**Authors:** Stefania Capuccio, Caterina Cocuzza, Grazia Letizia Di Marco, Alessandra Scamporrino, Salvatore Piro, Maurizio Russello

**Affiliations:** 1 Department of Clinical and Experimental Medicine, University of Catania, 95122 Catania, Italy; stefania.cap@hotmail.com (S.C.); salvatore.piro@unict.it (S.P.); 2 Department of Medicine and Surgery, “Kore” University of Enna, 94100 Enna, Italy; alessandra.scamporrino@unikore.it; 3 Liver Unit, National and High-Specialization Hospital Trust Garibaldi-Nesima, 95122 Catania, Italy; ccocuzza@arnasgaribaldi.it (C.C.); gdimarco@arnasgaribaldi.it (G.L.D.M.)

**Keywords:** dietary patterns, liver fibrosis, MASLD, MASH, nutrition, oxidative stress, insulin resistance, gut–liver axis, inflammation, metabolic syndrome, hepatokines, precision medicine

## Abstract

Metabolic dysfunction-associated steatotic liver disease (MASLD) represents the hepatic manifestation of systemic metabolic dysfunction and has emerged as one of the leading causes of chronic liver disease worldwide. Its pathogenesis is complex and multifactorial, involving insulin resistance, altered lipid metabolism, mitochondrial dysfunction, oxidative stress, and chronic low-grade inflammation. Beyond these classical mechanisms, growing evidence highlights the central role of modifiable lifestyle-related factors, including chronic positive energy balance, high intake of fructose and saturated fats, ultra-processed foods, physical inactivity, sleep disruption, and environmental exposures such as endocrine-disrupting chemicals and air pollution, which have been associated with the activation of lipogenic and proinflammatory pathways in preclinical and observational studies. Conversely, protective dietary patterns, particularly the Mediterranean diet, together with regular physical activity, exert hepatoprotective metabolic and anti-inflammatory effects. An extensive literature search was conducted across the PubMed, Scopus, Cochrane Library, and Embase databases, covering publications through June 2026. The review was conducted following the SANRA recommendations for narrative reviews. The inclusion criteria encompassed clinical trials, systematic reviews, meta-analyses, and international clinical practice guidelines. This review provides an integrated framework by linking in a single interpretative model the classical pathogenic pathways with emerging dietary, behavioral, and environmental determinants and with systemic interorgan communication networks involving hepatokines, adipokines, and myokines. Understanding MASLD as a multisystemic metabolic disease driven by multiple determinants has critical implications for the development of targeted preventive and therapeutic strategies aimed at reducing its global burden and prevalence.

## 1. Introduction

**Figure 1:**
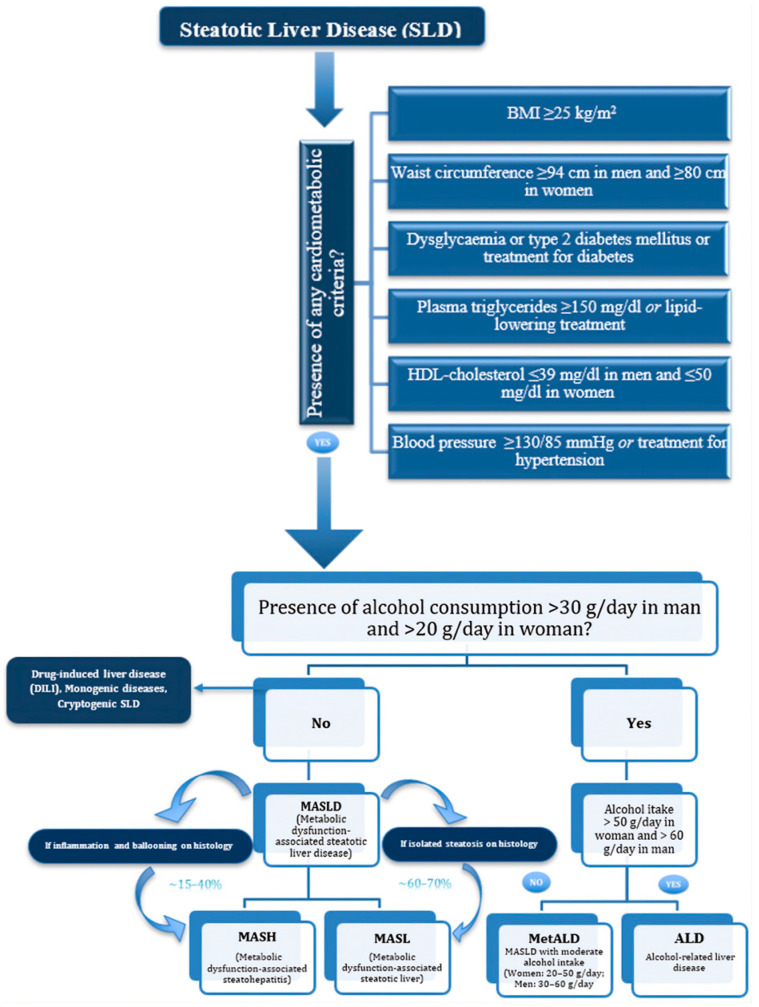
Classification and flow-chart for **steatotic liver disease** (SLD). MASLD is defined by hepatic steatosis plus ≥1 cardiometabolic risk factor, with 15–40% progressing to MASH. MetALD and ALD are distinguished by alcohol intake thresholds. Original illustration created by the author (*S. Capuccio*).

Metabolic dysfunction-associated steatotic liver disease (MASLD) is a condition closely linked to metabolic disorders such as obesity, type 2 diabetes mellitus (T2DM), dyslipidemia, and hypertension and has become one of the leading causes of liver-related morbidity and mortality worldwide [1]. The terminology was recently updated from NAFLD to MASLD through a global consensus involving 236 panelists from 56 countries, led by three major liver associations, the American Association for the Study of Liver Diseases (AASLD), the European Association for the Study of the Liver (EASL), and the Asociación Latinoamericana para el Estudio del Hígado (ALEH) [2]. Characterized by the accumulation of fat in the liver, MASLD encompasses a range of liver conditions, from simple steatosis to more advanced stages, including metabolic dysfunction-associated steatohepatitis (MASH), fibrosis, and cirrhosis. MASH is characterized by distinctive histological features, including hepatocellular ballooning and lobular inflammation. MASLD is now incorporated within the broader consensus definition of steatotic liver disease (SLD). This new classification of SLD also includes MASLD with moderate alcohol consumption (MetALD), alcohol-related liver disease (ALD), specific etiologies like drug-induced or monogenic steatotic liver diseases, and cryptogenic SLD [3] (Figure 1).

**Figure 2:**
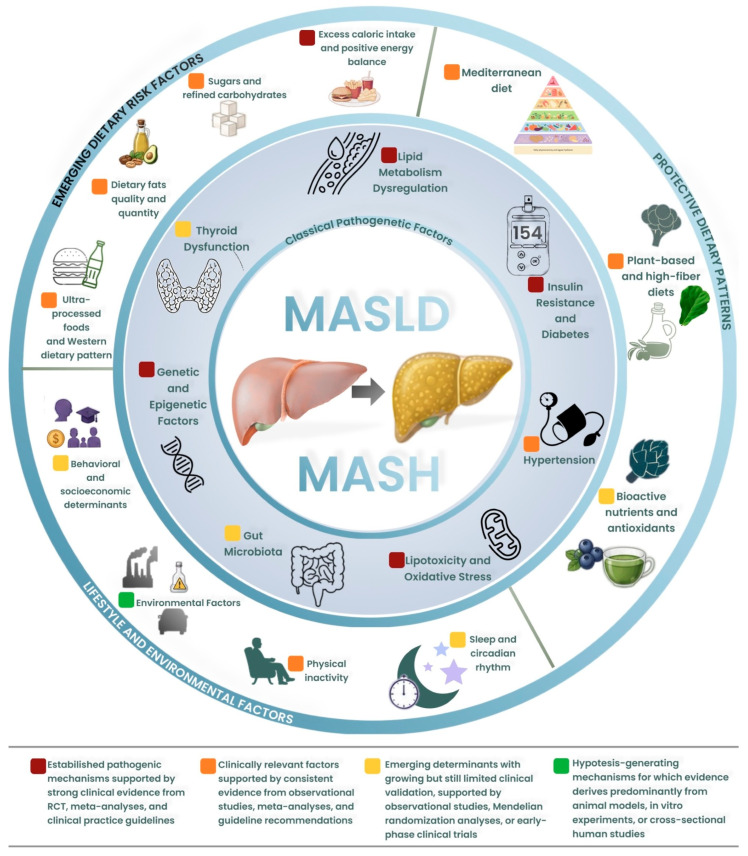
Pathophysiology of MASLD: a multi-layered and integrative model. Concentric rings depict classical pathogenetic mechanisms (inner), dietary and lifestyle/environmental determinants (middle), and conceptual framework (outer). Color coding reflects evidence strength. Original illustration created by the author (*S. Capuccio*).

The pathogenesis of MASLD is complex and multifactorial, involving genetic, environmental, and metabolic factors that interact to promote liver fat accumulation, insulin resistance (IR), and inflammation. Beyond these classical factors, a growing body of evidence highlights the role of additional modifiable determinants in the pathogenesis and progression of MASLD, including dietary risk factors, as well as lifestyle and environmental factors; conversely, protective dietary patterns such as the Mediterranean diet (MD), plant-based and high-fiber diets, and bioactive nutrients with antioxidant properties have emerged as potential modulators of disease risk and progression (Figure 2).

Although several recent reviews have addressed individual aspects of MASLD pathophysiology, most have focused on single pathogenic domains. To date, no comprehensive review has systematically integrated these classical and emerging mechanisms within a unified interpretative framework that also incorporates interorgan communication networks and modifiable lifestyle determinants. This review addresses this gap by proposing that MASLD should be understood not as a liver-restricted condition, but as a systemic metabolic disease in which hepatic, adipose, muscular, intestinal, and neuroendocrine dysfunctions converge through shared molecular pathways, including lipotoxicity, chronic inflammation, mitochondrial dysfunction, and organokine dysregulation, to drive disease onset and progression [4].

Furthermore, this review incorporates currently prominent concepts that are reshaping the MASLD landscape, including the recognition of distinct metabolic phenotypes and clinical heterogeneity of the disease, the emerging role of ferroptosis, cellular senescence, and extracellular vesicles in disease progression, the application of artificial intelligence (AI) and multi-omics approaches to diagnosis and risk stratification, and the development of precision medicine strategies for phenotype-guided therapeutic selection [5,6].

Understanding these interconnected mechanisms is essential to build an integrated model of disease prevention and management, capable of informing risk stratification, guiding phenotype-tailored therapeutic strategies, and ultimately reducing the global burden of MASLD and its hepatic and extrahepatic complications.

## 2. Methods

This narrative review was conducted following the Scale for the Assessment of Narrative Review Articles (SANRA) recommendations. A comprehensive literature search was performed across PubMed, Scopus, Embase, and the Cochrane Library, covering publications from inception through to June 2026.

The search strategy employed combinations of the following terms: ‘MASLD’, ‘MASH’, ‘NAFLD’, ‘NASH’, ‘metabolic dysfunction-associated steatotic liver disease’, ‘pathophysiology’, ‘insulin resistance’, ‘oxidative stress’, ‘lipotoxicity’, ‘gut microbiota’, ‘Mediterranean diet’, ‘ultra-processed foods’, ‘physical activity’, ‘environmental pollutants’, ‘endocrine disruptors’, ‘ferroptosis’, ‘cellular senescence’, ‘hepatokines’, ‘organokines’, ‘extracellular vesicles’, ‘precision medicine’, ‘artificial intelligence’, ‘multi-omics’, and ‘fibrosis’.

The inclusion criteria encompassed peer-reviewed original research articles, clinical trials, systematic reviews, meta-analyses, and international clinical practice guidelines published in English. Editorials, commentaries, case reports, and conference abstracts without full-text availability were excluded.

The initial search yielded approximately 1200 records; after screening by title and abstract and full-text evaluation for relevance and quality, 178 articles were included in the final review. Given the narrative nature of this review, no formal systematic quality assessment of individual studies was performed; however, priority was given to meta-analyses, randomized controlled trials, and guidelines from major scientific societies. The level of evidence supporting each mechanism discussed is indicated throughout the text and in the summary tables.

## 3. Overview of MASLD and Its Global Impact

MASLD has an estimated prevalence in the global population of approximately 30–38% [7]. Over the last 30 years, the global prevalence of MASLD has risen significantly, climbing from 17.6% in 1990 to 23.4% in 2019, with an average annual increase of approximately 1.0%. The prevalence of MASLD varies widely across regions, ranging from 25.1% in Western Europe to 44.4% in Latin America (LA), with the highest rates in LA and the Middle East/North Africa (MENA). MASLD is especially common in overweight or obese individuals, affecting about 50% globally and approximately 65–70% of those with T2DM, with a MASH prevalence of 32–35% in this population [8]. The global prevalence of MASH is approximately 5%, with regional differences: LA leads at 7.1%, followed by MENA (5.9%) and South Asia (5.4%), while Western Europe has the lowest rate at 4.0% [9]. In 2019, the global incidence of MASLD was estimated at 4.9%, with notable variation across countries: 4.3% in the USA, 5.1% in China, and 7.4% in Sri Lanka, among others. Incidence rates have risen sharply, increasing by nearly 60% from 3.7% in 1994–2006 to 5.9% in 2010–2014, raising significant concern in recent years [10]. While many individuals with MASLD remain asymptomatic, the disease has significant potential for progression, leading to more severe liver conditions, such as MASH, fibrosis, liver failure and hepatocellular carcinoma (HCC). Hepatic steatosis is a key feature of MASLD, defined by the presence of liver fat accumulation in combination with at least one cardiometabolic risk factor (Figure 1) [3].

MASLD can be classified into two forms based on histological features: MASL and MASH. MASL is defined by a combination of macrovesicular and microvesicular steatosis affecting at least 5% of hepatocytes. In 15–40% of patients, the condition advances to MASH, which is marked by hepatic steatosis (≥5% of hepatocytes), inflammation, and hepatocyte injury (ballooning), with or without fibrosis. MASH carries a higher risk of progression to cirrhosis, liver failure, and HCC [11,12].

Given its rising prevalence with serious long-term complications, MASLD is becoming a leading cause of liver transplantation in the United States and Europe, with MASH increasing from 9% to 28% of liver transplant indications in the US between 2000 and 2022 [13], and a major contributor to global healthcare costs [14]. The economic burden of MASLD is expected to rise in parallel with the global prevalence of metabolic disorders [15]. Therefore, understanding the pathogenesis of MASLD and developing effective therapeutic and preventive interventions are of critical importance to reduce its impact on public health.

## 4. Classical Pathophysiological Mechanisms of MASLD: A Comprehensive Survey

Dysregulation of lipid metabolism is a key driver of fatty liver disease, with MASLD being primarily characterized by the accumulation of triglycerides (TAGs) within hepatocytes. Previous research has shown that reducing triglyceride lipase levels can significantly decrease the risk of high-fat diet-induced MASH in mice [16].

The pathogenesis of MASLD is explained by the “multiple hits” theory. The initial “hit” involves hepatic TAGs overload, driven primarily by IR, lipotoxicity, and increased de novo lipogenesis (DNL). Subsequent events, including oxidative stress (OS), metabolic inflammation, endoplasmic reticulum stress, impaired autophagy, and signals from the intestinal microbiota, collectively contribute to a series of “parallel, multiple hits” that exacerbate liver damage [17] These mechanisms do not operate in isolation but form an interconnected pathophysiological network in which IR, lipotoxicity, OS, and inflammatory signaling mutually reinforce each other, creating self-perpetuating cycles that drive disease progression (Table 1).

MASLD arises from an imbalance in hepatic energy metabolism. When there is an excess intake of carbohydrates and fats, the liver’s capacity to oxidize these substrates becomes overwhelmed, leading to their storage as TAGs. Under normal conditions, insulin facilitates the glucose uptake in hepatocytes, enhances glycogen synthesis, and suppresses gluconeogenesis. However, in the context of IR, hepatocytes redirect excess glucose into lipogenic pathways, intensifying the TAGs accumulation that characterizes MASLD [35]. The typical paradigm of MASLD is primarily associated with obesity, T2DM, IR, and other components of metabolic syndrome. Consequently, MASLD and MASH occurring in the absence of overweight or obesity, as determined by these anthropometric measurements, are now referred to as “lean MASLD” [36]. Despite a leaner phenotype, these patients can still progress to MASH, advanced fibrosis, and adverse hepatic outcomes, underscoring that MASLD is not exclusively a disease of obesity [19].

### 4.1. MASLD and Lipid Metabolism

Lipid metabolism is crucial for processing dietary lipids and fatty acids (FAs), ensuring energy supply to organs. FAs are essential for energy production, membrane integrity, and signaling, but excessive levels cause lipotoxicity, leading to cellular stress, dysfunction, and death through detergent-like effects, acid-base imbalance, and toxic bioactive lipids like ceramides and diacylglycerols [37]. Disrupted lipid metabolism is central to MASLD [38]. Lipid overload and OS impair mitochondrial function, promote lipotoxicity, and amplify cytokine-mediated liver damage [39]. The main lipid species involved in liver lipid metabolism and lipotoxicity include TAGs, free cholesterol (FC), saturated fatty acids (SFA), monounsaturated fatty acids (MUFAs), polyunsaturated fatty acids (PUFAs), lysophosphatidylcholine (LPC), and sphingolipids, particularly ceramides [40]. MASLD livers exhibit increased SFAs, MUFAs, and *n*-6 PUFAs, with reduced *n*-3 PUFAs, resulting in an imbalanced *n*-6/*n*-3 PUFA ratio [41]. Although hepatic TAG accumulation is an initial step in MASLD, it is now considered protective against lipotoxicity, as impaired TAG synthesis shifts lipid accumulation toward FFAs, which exacerbate lipotoxic injury [23]. Severe dysregulation of hepatic cholesterol homeostasis has been reported in MASLD, leading to elevated levels of FC in the liver [42]. Cholesterol synthesis occurs in the endoplasmic reticulum (ER) and is tightly regulated by the enzyme HMGCR, which catalyzes the initial step in cholesterol biosynthesis [43]. FC accumulation is driven by increased HMGCR and SREBP-2 activity, with HMGCR expression elevated in steatosis and MASH livers, correlating with disease severity [44]. SFAs cause damage across various liver cell types, including hepatocytes, cholangiocytes, Kupffer cells (KCs), and hepatic stellate cells (HSCs) [23]. MUFAs are less harmful but still contribute to steatosis [45]. *N*-3 PUFAs protect against MASH [46], while *N*-6 PUFAs promote inflammation and disease advancement [47]. An *n*-6/*n*-3 imbalance accelerates MASLD progression [48]. LPC, elevated in MASH, drives SFA-induced lipotoxicity, damages cholangiocytes, and promotes fibrosis via hepatic stellate cell activation [49]. Ceramides in MASLD impair β-oxidation, increase ROS, and disrupt autophagy, leading to inflammation and apoptosis [50]. Emerging evidence also implicates ferroptosis, a form of iron-dependent regulated cell death driven by lipid peroxidation, as an additional mechanism of hepatocyte injury in MASLD [51].

### 4.2. Insulin Resistance and Diabetes

Insulin regulates glucose homeostasis by targeting the liver, muscle, and adipose tissue [52]. IR impairs cellular responsiveness, leading to hyperglycemia, increased FFA release, and lipid accumulation. Lipotoxicity exacerbates IR by activating inflammatory pathways like JNK and NF-κB, disrupting insulin signaling and enhancing OS [53]. In white adipose tissue (WAT), IR disrupts lipogenesis and lipolysis, causing adipocyte hypertrophy and increased cytokine production, which impairs insulin signaling and promotes inflammation. Adipokine imbalances, such as leptin resistance and reduced adiponectin, worsen IR by impairing fatty acid oxidation. In skeletal muscle, IR impairs GLUT4-mediated glucose uptake, with lipid intermediates like DAGs and ceramides disrupting insulin signaling. In the liver, excess FFAs and DNL contribute to steatosis and activate inflammatory pathways that inhibit insulin signaling [54]. The convergence of IR, lipotoxicity, and inflammation across these three tissues drives the progression from MASLD to MASH, with mitochondrial dysfunction and ER stress amplifying hepatocellular injury and fibrosis [35]. This multi-organ involvement underscores the systemic nature of MASLD. A pilot study in Italy found that patients with T2DM and MASLD with significant fibrosis had higher HbA1c levels compared to those with simple steatosis, highlighting the impact of fibrosis on glycemic control [22]. Conversely, poor glycemic management may contribute to the progression of MASLD. Supporting this, a study involving 713 biopsy-confirmed MASLD/MASH patients found that 49% had a concurrent diagnosis of diabetes [55].

### 4.3. Hypertension

Arterial hypertension is both a common comorbidity and an important pathogenic factor that contributes to disease progression in MASLD [56]. Blood pressure ≥ 130/85 mmHg or the use of antihypertensive medication is one of the five cardiometabolic criteria required to define MASLD in the presence of hepatic steatosis [12].

Hypertension is highly prevalent among patients with MASLD, with estimates ranging from 40% to over 80% depending on the population studied [26]. In a Swedish cohort of patients with MASLD, hypertension was present in 83.6% of cases, making it the second most prevalent cardiometabolic condition after overweight and obesity [26]. Several mechanisms explain how hypertension contributes to MASLD pathogenesis. Angiotensin II, the main effector of the renin–angiotensin–aldosterone system (RAAS), acts through the AT1 receptor to promote hepatic IR, OS, inflammation, and fibrogenesis. In transgenic animal models with RAAS overactivation, marked hepatic steatosis develops and progresses to steatohepatitis and fibrosis through increased production of reactive oxygen species and lipid peroxidation [57]. Beyond the RAAS, additional hormonal mediators contribute to the hypertension–MASLD interplay. Aldosterone promotes hepatic stellate cell activation and fibrogenesis independently of angiotensin II, while endothelin-1 exacerbates sinusoidal vasoconstriction and portal hypertension. Furthermore, high dietary sodium intake has been independently associated with MASLD prevalence in population-based studies, likely through mechanisms involving endothelial dysfunction, increased OS, and activation of pro-inflammatory pathways [29,30]. Chronic hypertension promotes hepatic inflammation through immune and neuroendocrine pathways, facilitating the transition from simple steatosis to MASH, creating a self-perpetuating vicious cycle [58].

In addition, hypertension exacerbates hepatic OS and impairs nitric oxide synthase activity enhancing vasoconstriction, with deleterious effects on both the liver and the cardiovascular system [58].

Zhou et al., in a large prospective multicenter study including three cohorts (UK Biobank, *n* = 107,316; VCTE-Prognosis, *n* = 8169; and Paired Liver Biopsy, *n* = 1670), showed that hypertension was independently associated with adverse long-term outcomes, including all-cause mortality, cardiovascular events, and liver-related events (adjusted HR 1.30, 95% CI 1.26–1.33), progression of liver stiffness (adjusted HR 1.57, 95% CI 1.30–1.90), and histological fibrosis progression (adjusted HR 1.41, 95% CI 1.12–1.78) [26]. These findings identify hypertension as a major modifiable risk factor for fibrosis progression in MASLD.

Recently, a study involving 35,988 patients with MASLD showed that treatment with angiotensin-converting enzyme inhibitors or angiotensin receptor blockers, compared with calcium channel blockers, was associated with lower all-cause mortality (HR 0.59, 95% CI 0.51–0.68), fewer major adverse liver outcomes (HR 0.70), and fewer major cardiovascular events (HR 0.82) [27].

### 4.4. Lipotoxicity and Oxidative Stress

Mitochondrial oxidative metabolism has been found to be twice as high in patients with MASLD compared to control individuals [59]. Saturated fatty acid accumulation disrupts mitochondrial dynamics, activating apoptotic pathways [16] and generating excessive ROS [23]. Accumulating evidence indicates that OS plays a central role in MASLD pathogenesis, acting as a mediator of cellular damage and inflammation [24]. OS is defined as an imbalance between the production of ROS and reactive nitrogen species (RNS) and the body’s ability to counteract their harmful effects through antioxidant defenses. In MASLD, mitochondrial dysfunction leads to pathological overproduction of ROS. Additionally, certain enzymes, such as NADPH oxidase, cyclooxygenase (COX), and CYP2E1, are upregulated in MASLD. Another key contributor is endoplasmic reticulum (ER) stress. The ER, a critical site for protein folding and modification, becomes overwhelmed in this condition, leading to the accumulation of misfolded proteins; this triggers an inflammatory response. The accumulation of ROS has severe downstream effects, including lipid peroxidation, protein oxidation, and DNA damage. Lipid peroxidation generates toxic byproducts, which worsen liver damage and inflammation through the activation of NF-κB signaling and the release of pro-inflammatory cytokines like TNF-α and IL-6 [60]. Reduced antioxidant defenses, including decreased enzymatic activity of key antioxidants like superoxide dismutase (SOD), catalase (CAT), and glutathione peroxidase (GPx), exacerbate OS and inflammation and fibrosis [25,61,62,63]. Chronic OS also inflicts DNA damage, contributing to genomic instability and mutations, which increases the likelihood of malignant transformation and the onset of HCC in patients with MASLD [64]. Mitochondria-associated membranes (MAMs), the contact sites between the ER and mitochondria, have recently emerged as critical regulators of lipid transfer, calcium signaling, and inflammatory responses in MASLD. Disruption of MAM integrity impairs lipid homeostasis and amplifies both ER stress and mitochondrial dysfunction, creating an additional layer of pathogenic complexity that connects lipotoxicity with OS and inflammation [65]. The interplay between lipotoxicity and OS creates a self-reinforcing cycle: lipotoxic intermediates impair mitochondrial function, generating excess ROS, which in turn amplify lipid peroxidation and further hepatocyte injury.

### 4.5. Gut Microbiota

The gut microbiota (GM) refers to the diverse community of microorganisms residing in the human gastrointestinal tract [66]. The liver and intestine are closely connected through the portal vein, with the gut–liver axis serving as the main communication pathway between the GM and the liver. This relationship is regulated by a complex network of metabolic, immune, and neuroendocrine signals. Tight junctions (TJs) in the gut epithelium serve as a barrier to bacteria and their metabolic products. When this barrier is compromised, pathogen-associated molecular patterns (PAMPs), such as lipopolysaccharides (LPS), activate NF-κB through toll-like receptors and nod-like receptors, leading to inflammation. PAMPs can also damage hepatocytes, activate stellate cells, and promote fibrosis, with Kupffer cells being particularly responsive to LPS [31]. The extent of liver damage is closely linked to the severity of gut dysbiosis, characterized by alterations in the composition of dominant phyla, such as Bacteroidetes and Firmicutes, including families like Ruminococcaceae, Lachnospiraceae, and Clostridiales. These bacteria produce short-chain fatty acids (SCFAs), which serve as an energy source for intestinal epithelial cells, modulate secondary bile acid metabolism, and enhance IgA production [67,68]. Gut dysbiosis increases ethanol production, activating TLRs in the liver, causing inflammation and OS and disrupting bile acid metabolism [69]. This leads to an imbalance in bile acid profiles, increased pathogenic bacteria, and impaired activation of key nuclear receptors (FXR and TGR5). FXR activation helps protect against dysbiosis, reduces TAGs levels, decreases IR, and modulates glucose metabolism [31]. Importantly, the composition and function of the gut microbiota are profoundly influenced by dietary quality. Diets rich in fiber, polyphenols, and fermented foods promote microbial diversity and SCFA production, whereas Western-type diets reduce microbial diversity, deplete butyrate-producing commensals, and increase intestinal permeability. This dietary modulation of the gut reinforces the concept that diet acts as a central upstream determinant of MASLD through both direct metabolic effects and indirect microbiota-mediated pathways [31,69].

### 4.6. Genetic and Epigenetic Factors

In recent years, numerous studies have focused on identifying gene variants and/or mutations that could be associated with MASLD. Stefan et al. identified three main MASLD phenotypes: one driven predominantly by hepatic genetic variants, characterized by high hepatic fat content without significant IR, and two metabolic phenotypes linked to hepatic DNL or adipose tissue dysfunction, respectively, each carrying distinct cardiometabolic risk profiles [19]. The Patatin-like Phospholipase Domain-Containing 3 (PNPLA3) is the most extensively and robustly characterized genetic variant with the largest effect size on hepatic fat content, MASH risk, and fibrosis progression [11], which influences hepatic lipid droplet remodeling and Very Low-Density Lipoprotein (VLDL) secretion [20,70]. The E167K variant of Transmembrane 6 Superfamily Member 2 (TM6SF2) alters VLDL synthesis and secretion and increases the risk of steatosis and fibrosis while paradoxically reducing cardiovascular risk through lower circulating lipid levels [65,67,68]. Reduced Membrane Bound O-Acetyltransferase Domain Containing 7 (MBOAT7) expression in obese patients is linked to liver damage and lipid metabolism alterations [18,21,71,72]. Cytochrome P450 2E1 (CYP2E1) overexpression promotes OS, inflammation, and insulin resistance but requires hepatic lipid accumulation to exert pathological effects [73,74]. Conversely, mutations in the Cell Death Inducing DFFA Like Effector B (CIDEB) and Hydroxysteroid 17-Beta Dehydrogenase 13 (HSD17B13) genes have a protective role, with CIDEB involved in lipid growth and storage in the liver [75].

Epigenetic modifications play a key role in disease progression: reduced Sirtuin 1 (SIRT1) expression, a regulator of OS and lipid metabolism, is associated with hepatic lipid accumulation through microRNA-34 (miR-34) overexpression [21,76]. Additionally, Peroxisome Proliferator-Activated Receptor Gamma Coactivator 1-Alpha (PGC-1α) methylation impairs mitochondrial biogenesis and promotes IR [21]. Polymorphisms like ENPP1 121 Gln and IRS-1 972 Arg, which affect insulin receptor activity, impair insulin signaling and are believed to contribute to MASLD progression [72]. Insulin-Like Growth Factor Binding Protein 2 (IGFBP2) hypermethylation is associated with both MASLD and T2DM [77], while in advanced stages, hypomethylation of TGF-β1, Collagen 1A1, and PDGF increases fibrosis risk [2,78,79].

### 4.7. Thyroid Dysfunction

Emerging evidence suggests that the thyroid plays a significant role in the development and progression of MASLD. The clinical relevance of the thyroid–liver axis has been validated by the FDA approval of resmetirom, a liver-directed selective THR-β agonist, in March 2024 for the treatment of non-cirrhotic MASH with fibrosis F2–F3 [80]. Zhang et al. identified hypothyroidism as an independent risk factor for MASLD, showing a clear dose–response relationship with disease severity [33]. Similar results were reported by Wang and colleagues in a large UK Biobank cohort, where hypothyroidism was associated with a significantly increased risk of MASLD [81].

A large meta-analysis by Zeng et al., including over 51,000 patients, confirmed that higher TSH and lower FT4 levels are associated with increased MASLD risk [82]. Additional cross-sectional studies reinforce these findings, demonstrating that both overt and subclinical hypothyroidism are independently associated with MASLD [83].

A particularly relevant aspect is the concept of intrahepatic hypothyroidism, a condition in which Thyroid hormones (THs) metabolism is locally impaired within the liver, even when circulating hormone levels remain within the normal range [84]. This concept remains largely based on preclinical evidence and should be considered an emerging hypothesis [34]. Alongside this, a second key element concerns the thyroid hormone receptor β (THR-β) [84]. Emerging evidence suggests that downregulation and impaired signaling of this receptor may represent an additional pathogenic mechanism contributing to MASLD progression, although this hypothesis requires further validation in large prospective human studies. In this context, the steatotic liver may be exposed to reduced THs signaling, but also progressively loses its ability to respond to it, creating a self-perpetuating cycle in which metabolic dysfunction reinforces itself [85].

The thyroid–liver axis represents a therapeutically actionable pathway, as demonstrated by Resmetirom’s efficacy [80], but the molecular mechanisms regulating local THs metabolism require further investigation in large prospective clinical studies.

### 4.8. Emerging Cellular Mechanisms: Ferroptosis, Cellular Senescence, Autophagy, and Extracellular Vesicles

**Figure 3:**
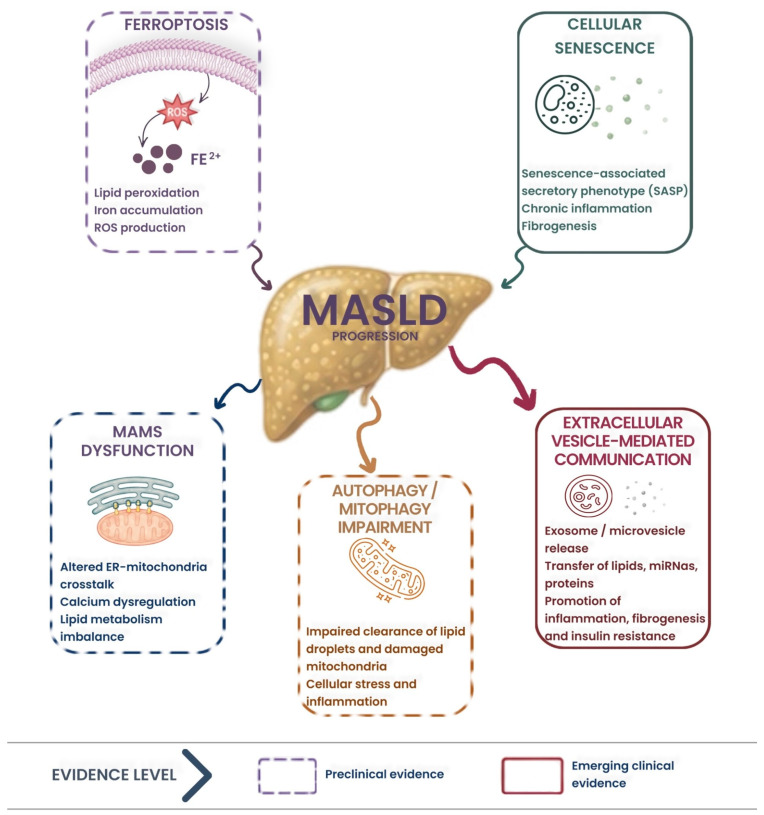
Emerging pathogenic pathways contributing to MASLD progression from steatosis to steatohepatitis, fibrosis and cirrhosis. Abbreviations: MAMS, Mitochondria-associated membranes; Fe^2+^, Ferrous iron; ROS, Reactive oxygen species; ER, *Endoplasmic Reticulum*. Original illustration created by the author (*S. Capuccio*).

Beyond the classical pathogenetic pathways, several cellular mechanisms have been implicated in MASLD progression, although most evidence derives from preclinical models (Figure 3).

Ferroptosis, an iron-dependent form of regulated cell death driven by lipid peroxidation, has emerged as a mechanism in MASLD. Paleman et al. identified a subgroup of biopsy-proven MASLD patients with hepatic ferroptosis signature and demonstrated that pharmacological ferroptosis inhibition attenuated steatosis in murine models [86]. Wang characterized ferroptosis as a driver of transition from prefibrotic states to overt fibrosis [51].

Cellular senescence, characterized by irreversible cell-cycle arrest and senescence-associated secretory phenotypes (SASP), has been linked to MASLD severity. SASP components (IL-1β, IL-6, TGF-β) promote chronic inflammation and fibrogenesis in the hepatic microenvironment. June et al. demonstrated that senescence-associated secretomes from Hedgehog-deficient hepatocytes perpetuate senescence and drive MASLD progression [87].

Impaired autophagy and mitophagy contribute to MASLD by allowing the accumulation of dysfunctional mitochondria and lipid droplets. Mitophagy dysfunction impedes hepatic energy homeostasis and exacerbates metabolic stress. The mitochondrial–autophagy axis represents an interconnected system in which impaired mitochondrial quality control amplifies OS, inflammation, and fibrotic remodeling [88,89].

Extracellular vesicles (EVs), nanoscale membrane-enclosed particles carrying bioactive cargoes including proteins and non-coding RNAs, have emerged as critical mediators of intercellular communication in MASLD. Lipotoxic hepatocyte-derived EVs modulate hepatic stellate cell activation, promoting inflammatory and fibrotic processes. EV-derived miRNA profiling has identified signatures associated with at-risk MASH, suggesting potential as non-invasive diagnostic biomarkers. However, standardization of EV isolation and characterization methods remains a significant challenge for clinical translation [90,91].

### 4.9. Interorgan Communication and Organokines

**Figure 4:**
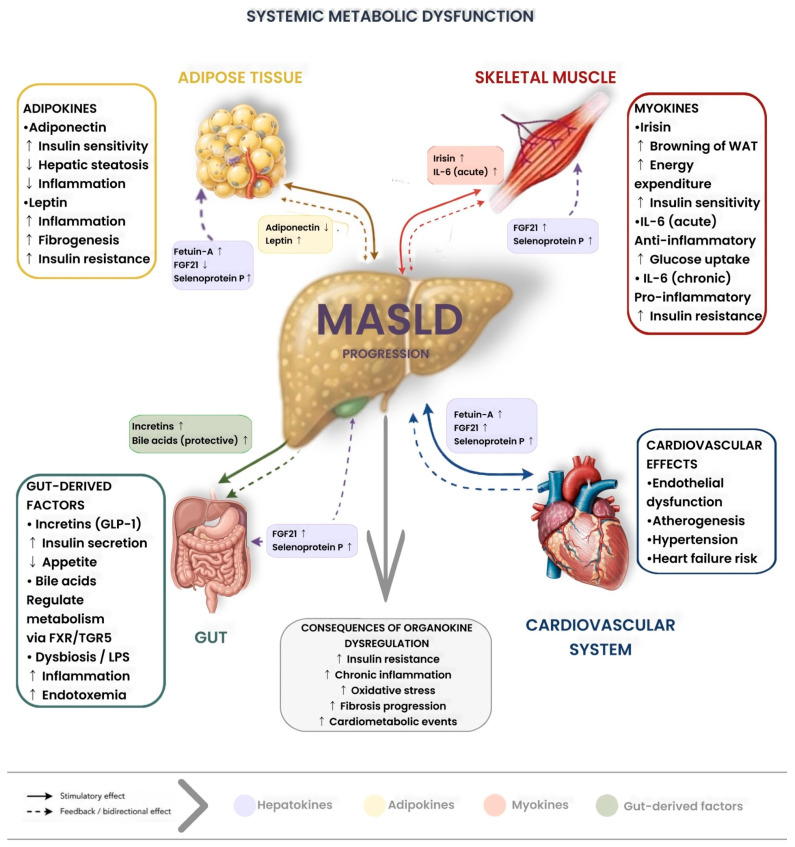
Interorgan crosstalk in MASLD: key organokines and their metabolic effects. Color coding indicates the origin of the different mediators involved in MASLD progression: hepatokines (purple), adipokines (yellow), myokines (pink), and gut-derived factors (green). Adipokines (adiponectin ↓, leptin ↑ with resistance, IL-6 ↑), hepatokines (FGF21 ↑, fetuin-A ↑), and myokines (irisin ↓, IL-6 ↑ during exercise) mediate bidirectional communication between adipose tissue, liver, and skeletal muscle, driving insulin resistance, lipotoxicity, inflammation, and fibrogenesis. Arrows indicate upregulation (↑) or downregulation (↓) in the context of MASLD. Abbreviations: IL-6, interleukin-6; FGF21, fibroblast growth factor 21. Original illustration created by the author (*S. Capuccio*).

MASLD is increasingly recognized as a systemic metabolic disease involving bidirectional communication between the liver, adipose tissue, skeletal muscle, gut, and cardiovascular system, mediated by organokines, signaling molecules secreted by specific organs that exert metabolic effects on distant tissues (Figure 4) [5,92].

Hepatokines, proteins secreted by the liver, play a central role in this interorgan crosstalk. Fetuin-A, elevated in MASLD, promotes adipose tissue inflammation and IR by acting as an endogenous ligand for TLR4 on macrophages. Selenoprotein P impairs insulin signaling in skeletal muscle and contributes to systemic IR. Conversely, FGF21, a hepatokine with pleiotropic metabolic effects, enhances fatty acid oxidation, improves insulin sensitivity, and reduces hepatic steatosis; its therapeutic potential is being explored through FGF21 analogues such as efruxifermin and pegozafermin [93,94,95].

Adipokine dysregulation is a hallmark of MASLD. Reduced adiponectin levels impair hepatic fatty acid oxidation and anti-inflammatory signaling, while leptin resistance promotes hepatic lipogenesis and fibrogenesis. Excess adipose tissue releases pro-inflammatory cytokines and recruits activated macrophages, sustaining systemic low-grade inflammation that amplifies hepatic injury [12].

Myokines, secreted by skeletal muscle during contraction, provide a mechanistic link between physical activity and hepatoprotection. Irisin enhances hepatic fatty acid oxidation and reduces lipogenesis, while exercise-induced IL-6 promotes anti-inflammatory macrophage polarization. Sarcopenia, increasingly recognized as a risk factor for MASLD progression, reduces myokine secretion and exacerbates metabolic dysfunction [96].

The gut–liver axis represents another critical interorgan communication pathway, with gut-derived metabolites (SCFAs, secondary bile acids, endotoxins) directly modulating hepatic metabolism and inflammation [31].

This interorgan perspective underscores that effective MASLD management requires addressing the systemic metabolic dysfunction that sustains it, providing the rationale for multitarget therapeutic approaches and multidisciplinary care models.

## 5. Emerging Dietary Risk Factors in MASLD

The development of MASLD is closely influenced by dietary composition, which affects hepatic lipid metabolism through several interrelated mechanisms—DNL, lipotoxicity, and OS—and alterations in the gut–liver axis [12,19].

Although excess caloric intake remains the main determinant of hepatic fat accumulation, growing evidence indicates that specific nutrients and overall dietary patterns can independently shape both the onset and progression of the disease, beyond their mere contribution to total energy intake (Table 2).

### 5.1. Excess Caloric Intake and Positive Energy Balance

A sustained positive energy balance is widely recognized as the main upstream driver of MASLD [106]. Lipids begin to accumulate ectopically in the liver, marking the onset of hepatic steatosis and initiating a cycle of progressive metabolic impairment [107]. Some studies show that hepatic TAGs derive mainly from increased FFAs flux due to IR (~60%), followed by DNL (~30%) and dietary fat (~10%). Among these, DNL is the most strongly upregulated pathway in MASLD, contributing disproportionately more to affected individuals compared with healthy subjects [12,19,106]. Disease progression is mainly driven by lipotoxic intermediates (FC, saturated fatty acids, diacylglycerols, ceramides), which induce mitochondrial dysfunction, OS, hepatocyte injury, and activation of inflammatory and fibrogenic pathways [12]. The importance of energy imbalance is supported by strong clinical evidence: weight loss leads to dose-dependent histological improvement. Specifically, weight loss of ≥5% improves steatosis, ≥7% is associated with resolution of steatohepatitis, and ≥10% can achieve fibrosis regression, as consistently demonstrated across multiple clinical trials [11,108]. Taken together, these findings identify chronic caloric excess as the principal metabolic driver in MASLD.

### 5.2. Sugars and Refined Carbohydrates

Among dietary macronutrients, sugars and refined carbohydrates have emerged as particularly potent modulators of MASLD pathogenesis, exerting hepatotoxic effects that extend well beyond their caloric contribution.

Fructose, mainly derived from sugar-sweetened beverages and foods containing sucrose or high-fructose corn syrup, exerts a distinct hepatotoxic effect. Unlike glucose, it bypasses phosphofructokinase regulation and is not subject to insulin-mediated feedback, leading to unregulated hepatic uptake, ATP depletion, increased uric acid production, and activation of DNL via SREBP-1c and ChREBP [109]. Several studies have shown that excessive fructose consumption increases the risk of MASLD, MASH, and hepatic fibrosis [100,110]. In a prospective study by Fan et al., higher fasting serum fructose levels were significantly associated with a higher prevalence of MASLD, which increased progressively across fructose quartiles (27.0%, 25.0%, 37.4%, and 44.5%; *p* < 0.001). Furthermore, each standard deviation increase in fasting serum fructose was associated with a 60% higher risk of MASLD (OR 1.60; 95%, CI 1.36–1.88; *p* < 0.001) [99]. However, these findings derive from observational data and do not establish a causal relationship between fructose intake and MASLD. Beyond lipogenesis, as recently demonstrated by Tang et al. in over 210,000 UK Biobank participants, fructose promotes disease progression through oxidative and endoplasmic reticulum stress, disruption of gut barrier integrity with consequent endotoxemia, and microbiota-mediated amplification of endogenous acetaldehyde production, which activates hepatic stellate cells and promotes fibrogenesis [111].

Accordingly, the ADA Consensus Report, the ACG Clinical Guideline, and the ADA Standards of Care uniformly emphasize the restriction of refined carbohydrates, added sugars, and fructose-rich beverages as a key component of dietary management in MASLD [112].

### 5.3. Dietary Fats Quality and Quantity

Although dietary lipids account for approximately 10% of intrahepatic TAGs, the type of fatty acid determines their metabolic fate and pathogenic potential [41]. In a randomized trial conducted by Rosqvist et al., isocaloric overfeeding with saturated fats (palm oil) was shown to increase liver fat by 50% and circulating ceramides, whereas polyunsaturated fats did not induce any increase in hepatic fat content despite a comparable weight gain [101]. A study conducted by Luukkonen et al. confirmed these findings, showing that overfeeding with saturated fats increased intrahepatic TAGs by 55% compared with only 15% with unsaturated fats [102]. SFAs impair mitochondrial respiratory chain efficiency, generate reactive oxygen species, and act as precursors of ceramides and diacylglycerols, which activate JNK signaling, disrupt insulin signaling, and induce hepatocyte apoptosis [19,113]. Lipidomic analyses of human liver biopsies have confirmed that patients with MASLD exhibit elevated hepatic concentrations of C16:0 and C18:1 with reduced polyunsaturated species, which correlates with histological severity independently of the total fat content [114]. FC within hepatic lipid droplets is a critical lipotoxic mediator and a key determinant of MASH development and fibrogenesis [115]. In contrast, omega-3 PUFAs exert hepatoprotective effects through SREBP-1c suppression, PPARα activation, and anti-inflammatory modulation of macrophages; supporting this, a meta-analysis has confirmed significant reductions in transaminases and hepatic fat following omega-3 supplementation [116]. Accordingly, current guidelines from EASL–EASD–EASO and the ADA recommend reducing saturated and trans-fat intake while favoring unsaturated fats, particularly within a Mediterranean dietary pattern, as a cornerstone of MASLD management [3,117,118].

### 5.4. Ultra-Processed Foods and Western Dietary Pattern

Ultra-processed foods (UPFs), as defined by the NOVA classification system, have emerged as an independent dietary risk factor for MASLD, with pathogenic effects extending beyond their unfavorable macronutrient profile [103,104,119].

A recent meta-analysis conducted by Guo et al. showed that higher consumption of ultra-processed foods is significantly associated with an increased risk of adverse liver outcomes (pooled OR 1.58; 95% CI 1.34–1.86), including MASLD (OR 1.72; 95% CI 1.36–2.17), liver fibrosis (OR 1.31; 95% CI 1.08–1.59), and HCC (OR 1.35; 95% CI 1.03–1.76) [103]. These associations are derived from observational studies and should be interpreted as risk associations rather than established causal relationships. Mediation analyses consistently show that the association between UPFs intake and MASLD is only partially mediated by adiposity (approximately 21%) and overall poor diet quality, indicating additional pathogenic mechanisms that are independent of body fat accumulation [120]. In a multi-omics analysis within the UK Biobank, 34 plasma metabolites and 65 proteins were found to be significantly associated with UPFs intake, with enrichment in pathways related to lipid metabolism, immune activation, and inflammatory responses. Notably, the proteomic signature of UPF consumption was associated with an 84% higher risk of MASLD (HR 1.84; 95% CI 1.45–2.35) and a 49% higher risk of cirrhosis (HR 1.49; 95% CI 1.16–1.91) [104].

The mechanistic basis for these adiposity-independent effects is increasingly attributed to food additives in UPFs. Emulsifiers (CMC, polysorbate 80) alter gut microbiota composition, reduce microbial diversity, increase bacterial mucosal penetration, and promote intestinal inflammation [121,122], while artificial sweeteners such as sucralose deplete butyrate-producing commensals [123]. These alterations converge on increased intestinal permeability and endotoxemia, activating Kupffer and hepatic stellate cells and driving progression to steatohepatitis and fibrosis [12,124].

The Western dietary pattern, characterized by high consumption of red and processed meat, refined grains, sugar-sweetened beverages, and UPFs, along with low intake of fruits, vegetables, legumes, and whole grains, represents the overall dietary exposure most consistently associated with the risk and progression of MASLD [19,125]. While the Western dietary pattern encompasses the individual dietary risk factors listed above, its inclusion as a separate category is justified because it captures the cumulative and synergistic effects of these components when consumed together as a habitual dietary pattern, which may exceed the sum of their individual contributions.

A meta-analysis of 18 studies confirmed that the Western dietary pattern is significantly associated with an increased risk of MASLD [105].

The Second EASL-Lancet Commission identifies UPFs as a leading source of saturated fats and added sugars and recommends minimizing their consumption as part of MASLD prevention strategies [126].

## 6. Protective Dietary Patterns

### 6.1. Mediterranean Diet

Among the dietary patterns evaluated in MASLD, the Mediterranean diet (MD) is the one supported by the most consistent and robust evidence base. Across observational studies and interventional trials, adherence to this dietary pattern has been associated with lower hepatic fat content, improved insulin sensitivity, and reduced markers of liver injury. Considering this evidence, the MD is currently recommended as the preferred dietary approach for patients with MASLD by major scientific societies, including the EASL–EASD–EASO Clinical Practice Guidelines, the AASLD Practice Guidance, the AGA Clinical Practice Update, and the American Diabetes Association Consensus Report [8,97,107,117]. The MD exerts hepatoprotective effects through multiple complementary mechanisms: MUFAs reduce hepatic DNL and improve insulin sensitivity; omega-3 PUFAs downregulate SREBP-1c-mediated lipogenesis while promoting PPARα-dependent β-oxidation; and polyphenolic compounds (hydroxytyrosol, oleuropein, quercetin) mitigate NF-κB-driven inflammation and enhance antioxidant defences via Nrf2 activation [127,128,129,130]. A recent systematic review and meta-analysis of 37 randomized controlled trials conducted by Arita et al. demonstrated that adherence to the MD is associated with significant improvements in anthropometric and biochemical outcomes in patients with MASLD/MASH [131]. In line with these findings, a cross-sectional NHANES analysis of 2672 MASLD patients further demonstrated that high MD adherence was associated with a 34% lower risk of significant liver fibrosis (OR 0.662; 95% 0.660–0.663; *p* for trend < 0.0001), with the protective effect attenuating as the cardiometabolic burden increased [125]. A longitudinal analysis of 119,536 UK Biobank participants conducted by George et al. demonstrated that each 5-unit increase in the Modified Mediterranean Diet Score was associated with 19% lower odds of MASLD. Higher adherence was also associated with lower hospitalization rates for liver-related, cardiovascular, and renal disease, and with reduced all-cause mortality in individuals with MASLD (HR 0.94; 95% CI 0.90–0.98) [132].

### 6.2. Plant-Based and High-Fiber Diets

Dietary fiber intake and plant-based dietary patterns have emerged as independent protective factors against the development and progression of MASLD, with effects that extend beyond simple caloric displacement [8]. Dietary fiber exerts hepatoprotective effects through multiple complementary mechanisms. Soluble fibers are fermented into SCFAs (butyrate, propionate, acetate), which enhance intestinal barrier integrity, reduce endotoxemia, suppress NF-κB activation, and modulate lipogenesis via AMPK signaling, while insoluble fibers blunt postprandial glycaemic peaks, reduce insulin-driven DNL, and increase fecal bile acid excretion, influencing FXR/TGR5 signaling [133].

The International Multidisciplinary Expert Consensus recommends increasing intake of whole grains, legumes, plant-based proteins, and fruits and vegetables as a core strategy for MASLD prevention and management, while the ADA Consensus Report similarly supports dietary patterns rich in fiber and unsaturated fats [8,117]. However, evidence for effects on steatohepatitis and fibrosis remains limited, as most data derive from observational studies and surrogate endpoints rather than randomized trials with histological outcomes.

### 6.3. Bioactive Nutrients and Antioxidants

OS is widely recognized as a key mechanism in the progression from simple steatosis to steatohepatitis, providing the rationale for evaluating antioxidant therapies in MASLD. Among these, vitamin E (800 IU/day) is the most extensively studied [134]. In the PIVENS trial, it significantly improved histological features of MASH, including higher rates of steatohepatitis resolution and reductions in steatosis, inflammation, and hepatocyte ballooning, although without improvement in fibrosis [107]. A subsequent meta-analysis confirmed improvements in transaminases and MASH resolution, but no effect on fibrosis. Accordingly, current AASLD guidelines recommend vitamin E only in selected non-diabetic patients with biopsy-proven MASH, while highlighting potential safety concerns with long-term high-dose use [135]. In contrast, evidence for dietary polyphenols such as curcumin, resveratrol, and silymarin remains preliminary [136]. Although some trials report improvements in metabolic parameters and liver enzymes, results are heterogeneous and generally of a limited quality, with uncertainty regarding clinically meaningful outcomes. A major limitation across these compounds is poor oral bioavailability, which restricts their therapeutic potential and complicates dose standardization [137]. Overall, vitamin E retains a limited but defined role in clinical practice, whereas current evidence is insufficient to support the routine use of other antioxidant compounds in the management of MASLD [134,138].

## 7. Lifestyle and Environmental Factors

### 7.1. Physical Inactivity

Physical inactivity and sedentary behavior emerge as independent, dose-dependent risk factors for the development and progression of MASLD [139]. Replacing sedentary time or light-intensity physical activity with moderate-to-vigorous physical activity (MVPA) is associated with a significant reduction in MASLD risk. Conversely, even small reductions in MVPA, when substituted with sedentary behavior, are linked to greater increases in the likelihood of developing MASLD [140]. Objectively measured physical activity shows an inverse association with MASLD across all intensity levels, with higher-intensity activities providing greater hepatoprotective benefits. These associations are primarily mediated by improvements in IR and reductions in waist circumference, suggesting that enhancements in metabolic health represent the main mechanism through which physical activity promotes its protective effects on the liver [139]. Structured exercise interventions reduce hepatic steatosis, transaminase levels, and IR, with aerobic exercise emerging as an intervention that may provide greater hepatic benefits compared to resistance training alone, although high-intensity activity has demonstrated efficacy in improving steatohepatitis and fibrosis [8].

Current evidence supports personalized physical activity programs including at least 150 min of moderate intensity activity or 75 min of vigorous intensity activity per week, together with resistance training 2–3 times weekly. Importantly, even brief 10 min activity sessions contribute meaningfully to achieving recommended targets and also help prevent sarcopenia [8].

### 7.2. Sleep and Circadian Rhythm

Sleep deprivation and circadian misalignment represent emerging pathophysiological drivers of MASLD, acting through disruption of the gut–liver–brain axis [141]. Sleep deprivation worsens hepatic steatosis by promoting gut dysbiosis, enhancing hepatic inflammation via NF-κB activation, and amplifying OS-related damage [142].

Sleep duration shows a non-linear relationship with MASLD, with optimal hepatoprotective effects observed between 7.5 and 9.5 h per day, while both short and prolonged sleep durations are associated with an increased risk [143].

Sleep apnea emerges as the sleep characteristic with the strongest causal relationship with MASLD in Mendelian randomization analyses [144].

Circadian rhythm misalignment driven by aberrant light exposure is a modifiable determinant of MASLD: each additional hour of daylight >6000 lux is associated with a 9% risk reduction, whereas each additional 30 min of nighttime light >30 lux corresponds to a 22% risk increase [145].

Artificial light at night (ALAN) exposure, particularly blue-enriched wavelengths (460–480 nm), suppresses melatonin secretion, disrupts hepatic circadian clocks, and promotes IR, OS, and steatosis through NF-κB activation [146], while circadian dysregulation further induces gut dysbiosis, increased intestinal permeability, and imbalances in SCFAs and bile acids, synergistically exacerbating hepatic lipid accumulation and inflammation [142].

### 7.3. Behavioral and Socioeconomic Determinants

Socioeconomic determinants of health appear to play a significant and independent role in MASLD risk, disease progression, and clinical outcomes [147]. Food insecurity, defined as limited or uncertain access to nutritionally adequate food, is strongly associated with MASLD prevalence [148]. Socioeconomic disparities likely influence disease outcomes through several mechanisms, including unequal access to healthcare, differential environmental exposures, chronic stress, and limited resources to implement and sustain lifestyle changes [149].

Hispanic and Latino populations have the highest prevalence of MASLD in the United States, with annual increases exceeding those observed in other ethnic groups. This pattern is attributable to a high burden of cardiometabolic risk factors, genetic predisposition, food insecurity, limited access to healthcare, language barriers, and lower health literacy [150]. In contrast, Black individuals have the lowest prevalence of MASLD despite higher rates of obesity and diabetes, suggesting the presence of protective genetic or environmental factors that remain incompletely understood [8].

Depression and anxiety, which are more prevalent among MASLD patients [151], represent important barriers to lifestyle modifications; systematic screening with validated instruments (PHQ-9, GAD-7) and integration of mental health support are recommended [152]. Addressing these structural and psychosocial determinants requires multiprofessional care models, culturally tailored interventions, and public health policies aimed at reducing socioeconomic inequalities.

### 7.4. Environmental Factors

Endocrine-disrupting chemicals (EDCs) and air pollutants are increasingly recognized as emerging contributors to MASLD pathogenesis [153], although much of the current evidence derives from animal studies, mechanistic experiments, and cross-sectional human studies rather than prospective clinical trials. Chronic low-dose exposure to EDCs, including phthalates, bisphenols, polyfluoroalkyl substances (PFAS), and polychlorinated biphenyls (PCBs), has been associated with steatosis, MASH, and fibrosis in preclinical and observational studies. Proposed mechanisms include disruption of nuclear receptor signaling, particularly PPARγ, induction of gut dysbiosis and intestinal barrier impairment, mitochondrial and lysosomal injury, and alterations in lipid metabolism [153]. Phthalate exposure has been consistently associated with MASLD prevalence in several cross-sectional human studies. In particular, metabolites of bis(2-ethylhexyl) phthalate (DEHP) have shown the strongest and most reproducible associations [154].

Exposure to EDCs during early life may have particularly important effects through epigenetic mechanisms. Experimental studies in animal models have shown that prenatal exposure to bisphenol A alters the hepatic epigenome and increases susceptibility to adiposity and MASLD by interacting with nuclear hormone receptors and genes involved in lipid metabolism [152,154]. Epidemiological studies have also reported a higher prevalence of MASH in communities with elevated PCB exposure. In addition, prospective data suggest that higher serum PCB concentrations are associated with incident MASLD, liver injury, and an increased risk of advanced fibrosis [155]. Micro- and nanoplastics (MNPs) represent an emerging area of concern in environmental hepatotoxicology. However, it is important to acknowledge that the human evidence linking MNPs to MASLD remains limited and largely indirect, based primarily on in vitro and animal studies. Preclinical evidence suggests that MNPs may accumulate in tissues and exacerbate metabolic injury through direct toxicity, chemical leaching, and transport of other environmental contaminants, but these findings require confirmation in human studies [153]. Given the widespread presence of EDCs in plastics, electrical products, and cosmetics, further research and policies aimed at reducing environmental exposure are warranted.

Air pollution, particularly PM2.5, PM10, and nitrogen oxides (NO_2_, NOx), has been identified as an independent risk factor associated with MASLD in observational studies, acting through metabolic reprogramming; metabolomic studies have identified panels of 65–87 metabolites mediating this association [156].

These findings support the need of public health strategies focused on chemical regulation and air quality improvement to mitigate the metabolic impact of environmental exposures.

## 8. Clinical and Preventive Implications

### 8.1. Risk Stratification and Early Identification

The medical and scientific community is investing considerable efforts in the development and validation of non-invasive tests (NITs) aimed at the early identification of patients with MASLD/MASH who are at increased risk of progression to advanced stages of liver disease. Consequently, this area of research is continuously evolving.

The Fibrosis-4 Index (FIB-4) represents the most cost-effective, evidence-based first-line strategy for risk stratification in primary care and diabetes settings. It is calculated using age, aspartate aminotransferase (AST), alanine aminotransferase (ALT), and platelet count. FIB-4 screening should be universally implemented in all adults with T2DM or prediabetes associated with cardiometabolic risk factors, even in the presence of normal aminotransferase levels [112].

Importantly, longitudinal evidence from retrospective studies with paired liver biopsies has shown that increases over time in NITs such as AST to Platelet Ratio Index (APRI), FIB-4, and NAFLD Fibrosis Score (NFS) are significantly associated with one-stage fibrosis progression. FIB-4 and NFS demonstrate high negative predictive values (~90%) but suboptimal positive predictive value for advanced fibrosis. Longitudinal data support their ability to predict liver-related mortality, HCC, cardiovascular mortality, and progression to cirrhosis risk, although their accuracy for monitoring therapeutic response remains insufficiently validated [3]. Patients with FIB-4 ≥1.3 should undergo second-line assessment using vibration-controlled transient elastography (VCTE) or the Enhanced Liver Fibrosis test (ELF). Liver stiffness measurement (LSM) values between 10 and 20 kPa, or ELF values between 9.2 and 11.3, may indicate consideration of pharmacological therapy after exclusion of cirrhosis [157].

The Agile 3+ and Agile 4 scores, which integrate LSM with clinical parameters, demonstrate superior prognostic performance (area under the receiver operating characteristic curve, AUROC 0.87–0.91) compared with LSM alone or conventional fibrosis scores. These tools enable more robust risk stratification, clearly distinguishing low-risk from high-risk patients [158]. Emerging evidence suggests that polygenic risk scores (PRS) incorporating variants in PNPLA3, TM6SF2, and HSD17B13 independently predict fibrosis progression and liver-related events, with the PNPLA3 G/G genotype being associated with significantly higher liver stiffness; however, the current practice guidelines do not yet recommend routine genetic testing for MASLD screening [159,160].

AI and machine learning (ML) approaches are increasingly being applied to MASLD diagnosis and risk stratification. Machine learning approaches integrating demographic, metabolic, lipid, and biochemical biomarkers achieve an accuracy of 79.59% for the prediction of MASLD and 86.07% for the prediction of fibrosis, with age, BMI, and insulin emerging as significant predictors in both models [161]. Deep learning algorithms applied to imaging data (ultrasound, CT, MRI) have shown promising results for automated steatosis quantification and fibrosis staging, potentially reducing operator dependency and improving diagnostic reproducibility. Furthermore, AI-driven integration of multi-omics data is emerging as a powerful tool for identifying novel biomarker panels and predicting individual disease trajectories [162].

Emerging biomarkers, including CK-18, FGF21, and multi-omic signatures, may improve phenotypic classification and risk stratification, although prospective validation is still required [163].

### 8.2. Lifestyle-Based Prevention and Precision Counseling

Lifestyle modification remains the foundation of both prevention and treatment of MASLD. Sustained weight loss of 7–10% has been consistently associated with significant histological improvement, including resolution of steatohepatitis and regression of fibrosis [164]. Limiting free sugars, particularly fructose, decreases hepatic DNL and improves both metabolic and liver-related outcomes [12,152].

Diet represents a critical modifiable determinant that should be systematically addressed alongside physical activity, sleep optimization, and behavioral interventions as part of comprehensive MASLD management.

Regular physical activity is fundamental. Tailoring exercise programs to each patient’s physical capacity, preferences, and confidence is essential to promote long-term adherence [8]. Treatment strategies should be adapted to the underlying MASLD phenotype. Patients in whom disease is primarily driven by excess caloric intake and increased DNL benefit most from dietary modification and increased physical activity [19].

When lifestyle measures are insufficient to achieve meaningful weight loss, bariatric surgery and incretin-based therapies, such as GLP-1 receptor agonists, represent effective options [165,166]. In contrast, patients with lean MASLD or predominant adipose tissue dysfunction may benefit more from emerging therapies such as PPAR agonists, dual or triple incretin receptor agonists, THR-β agonists, and FGF-21 analogues [19]. Genetic variants such as PNPLA3 and TM6SF2 are associated with a higher risk of steatohepatitis, advanced fibrosis, cirrhosis, and HCC. Their presence may justify closer monitoring and earlier consideration of pharmacological treatment [19].

Because MASLD is a multisystemic disorder, optimal management requires a multidisciplinary approach involving hepatologists, endocrinologists, dietitians, psychologists, physiotherapists, and social workers.

Alcohol intake should be minimized in all patients and avoided completely in those with moderate or advanced fibrosis, given its potential to accelerate liver injury [167].

### 8.3. Translational Opportunities for Biomarkers and Therapies

The therapeutic landscape of MASLD has evolved rapidly with the FDA approval of resmetirom, a selective thyroid hormone receptor β agonist, and semaglutide 2.4 mg, a GLP-1 receptor agonist, for patients with non-cirrhotic MASH and fibrosis (stages F2-F3) [80,168]. Resmetirom 100 mg has demonstrated efficacy in the phase 3 MAESTRO-NASH trial, achieving MASH resolution in 29.9% of patients and fibrosis improvement by ≥1 stage in 25.9% after 52 weeks of treatment (vs. 9.7% and 14.2% with placebo, respectively; *p* < 0.001 for both) [80]. Semaglutide 2.4 mg has also demonstrated substantial efficacy, achieving MASH resolution in 63% of patients and fibrosis improvement in 37% after 72 weeks of treatment [168].

The mechanistic basis for the efficacy of these agents can be understood through the pathophysiological framework presented in this review.

Resmetirom directly addresses the thyroid–liver axis dysfunction, restoring THR-β–mediated lipid metabolism and mitochondrial function. GLP-1 receptor agonists target multiple pathogenic nodes, including IR, adipose tissue dysfunction, organokine dysregulation, and hepatic lipotoxicity, thereby illustrating how therapeutic strategies aligned with specific pathophysiological mechanisms can achieve meaningful clinical outcomes. Among emerging therapies, tirzepatide, a dual GIP/GLP-1 receptor agonist, has produced particularly encouraging results, with MASH resolution in 40–60% of treated patients and fibrosis improvement in approximately half of cases [169]. Survodutide, a dual glucagon/GLP-1 receptor agonist, has also demonstrated significant efficacy in the phase 2 trial, achieving histological improvement in MASH and fibrosis, with additional benefits on hepatic fat reduction and body weight [170].

Other agents in advanced clinical development include triple incretin receptor agonists, FGF21 analogues such as efruxifermin and pegozafermin, PPAR agonists such as lanifibranor, and fatty acid synthase inhibitors [171].

SGLT2 inhibitors have shown hepatoprotective effects in MASLD through mechanisms including reduction in hepatic fat content, improvement of insulin sensitivity, attenuation of OS, and modulation of autophagy. Meta-analyses of randomized trials have demonstrated significant reductions in ALT, hepatic steatosis indices, and body weight in patients with T2DM and MASLD treated with SGLT2 inhibitors. However, dedicated phase 3 trials with histological endpoints in MASH are still lacking, and their role in MASLD management remains to be fully defined [172].

Treatment selection should be individualized through shared decision-making, considering the patient’s cardiometabolic profile, presence of extrahepatic complications, therapeutic goals, treatment costs, and patient preferences.

Response to therapy can be monitored using non-invasive biomarkers, including a reduction of at least 30% in LSM or a decrease of at least 0.5 points in the ELF score [157].

Important unmet needs remain, including effective therapies for MASLD-related cirrhosis, for which no liver-directed pharmacological treatments are currently available, the identification of reliable predictive biomarkers of treatment response, and the lack of data defining the optimal duration of therapy [12].

Gut microbiome-targeted interventions, including fecal microbiota transplantation and next-generation probiotics (e.g., *Akkermansia muciniphila),* represent a promising area for personalized therapeutic strategies [173].

Overall, the convergence of novel metabolic therapies, microbiome modulation, genetic risk stratification, and non-invasive monitoring is ushering in a new era of precision medicine for MASLD, based on a comprehensive and multidisciplinary approach to patient care.

## 9. Future Directions

MASLD is currently the subject of intense research from pathogenic, diagnostic, and therapeutic perspectives. A better understanding of the mechanisms underlying disease heterogeneity is needed, with emphasis on the interplay among genetic variants, metabolic phenotypes, and environmental exposures in determining disease progression and treatment response.

A second major priority is the validation of non-invasive biomarkers for diagnosis, risk stratification, and treatment monitoring that can be implemented in primary care and in resource-limited settings.

Another important unmet need is the development of combination therapies capable of targeting multiple pathophysiological pathways simultaneously, with careful evaluation of their synergistic efficacy and safety.

There is also a pressing need for effective treatments for advanced stages of disease, particularly MASLD-related cirrhosis, for which no liver-directed pharmacological therapies are currently available.

Future priorities include the development of precision medicine approaches integrating genetic risk assessment (polygenic risk scores), metabolic phenotyping, gut microbiome profiling, and multi-omics technologies (metabolomics, lipidomics, transcriptomics, proteomics) for early identification of patients at risk of progression. AI applied to multi-omics datasets and imaging data holds promise for improving diagnostic accuracy and guiding phenotype-specific therapeutic selection.

Emerging cellular mechanisms, ferroptosis, cellular senescence, MAM dysfunction, extracellular vesicles, and mitophagy impairment represent promising therapeutic targets requiring clinical validation. Similarly, interorgan communication mediated by organokines offers opportunities for multitarget therapeutic development, although longitudinal studies are needed to define their prognostic utility.

Finally, attention to critical windows of environmental exposure and the implementation of upstream nutritional prevention policies are essential to address the metabolic determinants of MASLD at a population level [174].

## 10. Limitations

This review has several limitations inherent to its narrative design. Although an extensive literature search was performed across multiple databases following SANRA recommendations, the absence of a formal systematic methodology means that some relevant studies may have been inadvertently omitted.

The included studies are heterogeneous in design, ranging from randomized controlled trials and meta-analyses to animal studies and cross-sectional analyses. Several emerging mechanisms discussed, including ferroptosis, cellular senescence, MAM dysfunction, and environmental pollutants, are supported primarily by preclinical evidence, and their clinical relevance in human MASLD remains to be fully established.

## 11. Conclusions

**Figure 5:**
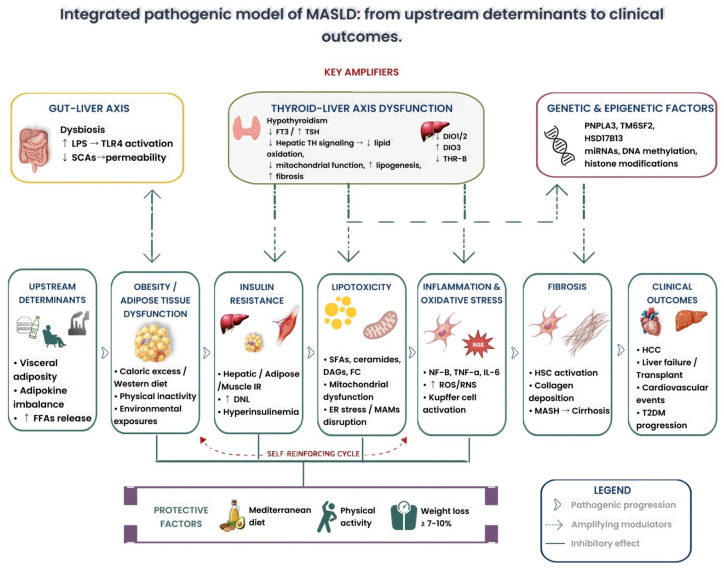
Integrated pathogenetic model of MASLD: from upstream determinants to clinical outcomes. This schematic provides a simplified overview of the complex interactions discussed throughout the manuscript, emphasizing that MASLD progression results from the convergence of multiple interconnected pathways rather than a single linear cascade. Abbreviations: FFAs, free fatty acids; DNL, de novo lipogenesis; SFAs, saturated fatty acids; DAGs, diacyglycerols; FC, free cholesterol; ER, endoplasmic reticulum; MAMs, mitochondria-associated membranes; NF-B, nuclear factor B; ROS, reactive oxygen species; RNS, reactive nitrogen species; HSC, hepati stellate cell; HCC, hepatocellular carcinoma; T2DM, type 2 diabetes mellitus; TH, thyroid hormone; T3, triiodothyronine; TSH, thyroid-stimulating hormone; DIO, deiodinase; THR-B, thyroid hormone receptor beta; ↑ increase; ↓ decrease. Original illustration created by the author (*S. Capuccio*).

MASLD represents the hepatic manifestation of a multisystem metabolic disease. The progression from simple steatosis to systemic metabolic dysfunction reflects a complex, synergistic pathophysiological network. Together, all these factors drive the transition from isolated steatosis to steatohepatitis, fibrosis, cirrhosis, HCC, and a broad range of extrahepatic complications. Notably, MASLD and MASH increase the risk of T2DM and cardiovascular disease through a bidirectional relationship: hepatic IR and atherogenic dyslipidemia promote cardiometabolic disorders, while diabetes and cardiovascular disease, in turn, accelerate hepatic inflammation and fibrosis progression [8,19,160] (Figure 5).

Modifiable determinants, including physical inactivity, unhealthy dietary patterns, UPFs intake, sleep deprivation, food insecurity, exposure to endocrine-disrupting chemicals, and air pollution, represent important prevention and therapeutic targets. However, it is essential to distinguish between established risk factors supported by strong clinical evidence (e.g., caloric excess, physical inactivity, saturated fat intake) and emerging determinants for which evidence remains preliminary (e.g., microplastics, endocrine disruptors). Addressing these factors extends beyond conventional lifestyle counseling and requires broader public health and policy interventions aimed at improving the built environment, food systems, chemical regulation, and access to healthcare.

The therapeutic landscape has evolved from an approach based exclusively on lifestyle modification to include FDA-approved agents such as resmetirom and semaglutide, as well as emerging precision medicine strategies involving incretin-based therapies, SGLT2 inhibitors, gut microbiome modulation, and phenotype-guided treatment selection.

The mechanistic understanding of how these therapies target specific pathophysiological nodes, from thyroid–liver axis dysfunction to IR, organokine dysregulation, and gut–liver axis impairment, provides a rational framework for individualized therapeutic selection.

The emerging recognition of MASLD as a systemic disease involving bidirectional interorgan communication through hepatokines, adipokines, and myokines, together with the identification of novel cellular mechanisms such as ferroptosis, cellular senescence, and extracellular vesicle-mediated signaling, opens new avenues for biomarker discovery and targeted therapeutic development.

Addressing the global burden of MASLD requires multidisciplinary models of care that integrate management of both hepatic and extrahepatic risk and the systematic implementation of evidence-based screening algorithms.

The future of MASLD management lies in recognizing disease heterogeneity, identifying and targeting the upstream metabolic, behavioral and environmental drivers of liver injury, and delivering individualized care that reflects the multisystem nature of this increasingly important public health challenge.

## Figures and Tables

**Table 1:** Classical pathophysiological mechanisms of MASLD.

Pathogenic Domain	Key Mechanisms	Principal Mediators	Evidence Level	References
Genetic Variants	Lipid droplet/VLDL defects	Risk-associated: PNPLA3, TM6SF2, MBOAT7; Protective: CIDEB, HSD17B13	Strong clinical evidence (GWAS, large cohorts)	Helsley et al. 2019 [18]; Stefan et al. 2025 [19]; Wang et al. 2025 [20]
Epigenetic Modifications	DNA methylation; miRNA dysregulation	↓ SIRT1; PGC-1α/TGF-β1 methylation changes	Emerging/ Preclinical evidence	Juanola et al. 2021 [21]
Insulin Resistance	Impaired hepatic/adipose/muscle signaling; ↑ FFAs/DNL	JNK, NF-κB; ↓ adiponectin, leptin resistance	Strong clinical evidence (RCTs, large cohorts)	Mantovani et al. 2022 [22]; Stefan et al. 2025 [19]; Tilg et al. 2026 [12]
Lipid Metabolism	↑ DNL; TAG/FC overload; altered PUFA (↓ *n*-3/↑ *n*-6)	SFAs, ceramides, DAGs	Strong clinical evidence (meta-analyses, human biopsies)	Fuchs et al. 2022 [16]; Iturbe-Rey et al. 2025 [23]
Oxidative Stress	Mitochondrial/ER dysfunction	↑ ROS/RNS; ↓ antioxidants; NF-κB → TNF-α, IL-6	Strong clinical evidence (human biopsies, interventional trials)	Masarone et al. 2018 [24]; Jomova et al. 2024 [25]
* Hypertension	RAAS overactivation; ↓ NO synthase; SNS activation; Na^+^-mediated endothelial dysfunction	↑ ROS, lipid peroxidation, Ang II, aldosterone, ET-1	Strong clinical evidence (large prospective cohorts)	Zhou et al. 2024 [26]; Ng Whet al. 2026 [27]; Hernández-Rubio et al. 2026 [28]; Liu et al. 2025 [29]; Van der Graaff et al. 2018 [30]
Gut Dysbiosis	↑ Permeability → bacterial translocation	LPS → TLR → NF-κB; ↓ SCFAs	Moderate clinical evidence (observational, mechanistic)	Milosevic et al. 2019 [31]; Tripathi et al. 2018 [32]
Thyroid Dysfunction	Intrahepatic hypothyroidism; THR-β ↓	↓ T3/THR-β; ↑ TSH	Moderate clinical evidence (meta-analyses, cross-sectional)	Zhang et al. 2022 [33]; Kuchay et al. 2024 [34]

The table summarizes the recognized pathogenic mechanisms implicated in the development and progression of MASLD. * Hypertension is listed in this table as both a cardiometabolic comorbidity and an active pathogenetic contributor to MASLD. Abbreviations: DNL, de novo lipogenesis; TAG, triacylglycerol; FC, free cholesterol; PUFA, polyunsaturated fatty acids; *n*-3/*n*-6, omega-3/omega-6 fatty acids; SFAs, saturated fatty acids; DAGs, diacylglycerols; FFAs, free fatty acids; JNK, c-Jun *N*-terminal kinase; NF-κB, nuclear factor kappa-light-chain-enhancer of activated B cells; RAAS, renin–angiotensin–aldosterone system; NO, nitric oxide; ROS, reactive oxygen species; SNS, sympathetic nervous system; Na^+^, sodium; Ang II, angiotensin II; ET-1, endothelin-1; ER, endoplasmic reticulum; RNS, reactive nitrogen species; TNF-α, tumor necrosis factor-alpha; IL-6, interleukin-6; LPS, lipopolysaccharide; TLR, toll-like receptor; SCFAs, short-chain fatty acids; VLDL, very low-density lipoprotein; PNPLA3, patatin-like phospholipase domain-containing 3; TM6SF2, transmembrane 6 superfamily member 2; MBOAT7, membrane-bound O-acyltransferase domain-containing 7; CIDEB, cell death-inducing DFFA-like effector B; HSD17B13, hydroxysteroid 17-beta dehydrogenase 13; miRNA, microRNA; SIRT1, sirtuin 1; PGC-1α, peroxisome proliferator-activated receptor gamma coactivator 1-alpha; TGF-β1, transforming growth factor-beta 1; THR-β, thyroid hormone receptor-beta; T3, triiodothyronine; TSH, thyroid-stimulating hormone; ↑ increase; ↓ decrease.

**Table 2:** Emerging dietary risk factors for MASLD.

Dietary Factor	Pathogenic Mechanism	Key Data	Study Design	References
Caloric excess	↑ FFAs, ↑ DNL, lipotoxic intermediates (FC, SFAs, DAGs, ceramides)	≥10% WL: 90% MASH resolution, 45% fibrosis regression (*p* < 0.001)	RCTs, prospective cohorts	Younossi et al. 2021 [97]; Vilar et al. 2015 [98]
Fructose/added sugars	Unregulated hepatic uptake; ↑ DNL; gut barrier disruption; HSC activation	OR 1.60 per SD ↑ fasting fructose (95% CI 1.36–1.88; *p* < 0.001)	Prospective cohort	Fan et al. 2024 [99]; Yki-Järvinen et al. 2021 [100]
Saturated fatty acids	↑ Ceramides/DAGs → JNK activation, ↓ insulin signaling	SFA overfeeding +50% liver fat vs. PUFAs (*p* < 0.05)	RCTs	Rosqvist et al. 2019 [101]; Luukkonen et al. 2018 [102]
Ultra-processed foods	Gut dysbiosis, ↑ intestinal permeability → endotoxemia	OR 1.72 MASLD (95% CI 1.36–2.17), OR 1.31 fibrosis (1.08–1.59)	Meta-analysis of observational studies	Guo 2025 et al. [103]; Zhao et al. 2025 [104]
Western dietary pattern	Lipotoxicity, ↑ IR, chronic inflammation, gut dysbiosis	OR 1.56 MASLD (95% CI 1.27–1.92; *p* ≤ 0.001)	Meta-analysis of observational studies	Hassani et al. 2021 [105]

The table summarizes the main emerging dietary risk factors implicated in the development and progression of MASLD. Abbreviations: FFAs, free fatty acids; DNL, de novo lipogenesis; WL, weight loss; OR, odds ratio; SD, standard deviation; DAGs, diacylglycerols; SFA, saturated fatty acid; PUFAs, polyunsaturated fatty acids; IR, insulin resistance; RCTs, randomized controlled trials; ↑ increase; ↓ decrease.

## Data Availability

No new data were created or analyzed in this study. Data sharing is not applicable to this article.

## Abbreviations

ALD, alcohol-related liver disease; ALAN, artificial light at night; ALT, alanine aminotransferase; AMPK, AMP-activated protein kinase; APRI, AST to platelet ratio index; AST, aspartate aminotransferase; AUROC, area under the receiver operating characteristic curve; BMI, body mass index; CAT, catalase; ChREBP, carbohydrate-responsive element-binding protein; CIDEB, cell death-inducing DFFA-like effector B; CMC, carboxymethylcellulose; COX, cyclooxygenase; CYP2E1, cytochrome P450 2E1; DAGs, diacylglycerols; DEHP, bis(2-ethylhexyl) phthalate; DNL, de novo lipogenesis; EDCs, endocrine-disrupting chemicals; ELF, Enhanced Liver Fibrosis test; ER, endoplasmic reticulum; EVs, extracellular vesicles; FC, free cholesterol; FAs, fatty acids; FFAs, free fatty acids; FGF21, fibroblast growth factor 21; FIB-4, Fibrosis-4 Index; FXR, farnesoid X receptor; GIP, glucose-dependent insulinotropic polypeptide; GLP-1, glucagon-like peptide-1; GM, gut microbiota; GPx, glutathione peroxidase; HbA1c, glycated hemoglobin; HCC, hepatocellular carcinoma; HMGCR, 3-hydroxy-3-methylglutaryl-CoA reductase; HR, hazard ratio; HSD17B13, hydroxysteroid 17-beta dehydrogenase 13; HSCs, hepatic stellate cells; IGFBP2, insulin-like growth factor binding protein 2; IR, insulin resistance; JNK, c-Jun *N*-terminal kinase; KCs, Kupffer cells; LA, Latin America; LPC, lysophosphatidylcholine; LPS, lipopolysaccharide; LSM, liver stiffness measurement; MAMs, mitochondria-associated membranes; MASL, metabolic dysfunction-associated steatotic liver; MASLD, metabolic dysfunction-associated steatotic liver disease; MASH, metabolic dysfunction-associated steatohepatitis; MBOAT7, membrane-bound O-acyltransferase domain-containing 7; MD, Mediterranean diet; MENA, Middle East and North Africa; MetALD, MASLD with moderate alcohol consumption; miRNA, microRNA; MNPs, micro- and nanoplastics; MUFAs, monounsaturated fatty acids; MVPA, moderate-to-vigorous physical activity; NAFLD, non-alcoholic fatty liver disease; NF-κB, nuclear factor kappa-light-chain-enhancer of activated B cells; NFS, NAFLD Fibrosis Score; NITs, non-invasive tests; NO, nitric oxide; Nrf2, nuclear factor erythroid 2-related factor 2; OR, odds ratio; OS, oxidative stress; PAMPs, pathogen-associated molecular patterns; PCBs, polychlorinated biphenyls; PDGF, platelet-derived growth factor; PFAS, polyfluoroalkyl substances; PGC-1α, peroxisome proliferator-activated receptor gamma coactivator 1-alpha; PNPLA3, patatin-like phospholipase domain-containing 3; PPARα, peroxisome proliferator-activated receptor alpha; PPARγ, peroxisome proliferator-activated receptor gamma; PRS, polygenic risk scores; PUFAs, polyunsaturated fatty acids; RAAS, renin–angiotensin–aldosterone system; RNS, reactive nitrogen species; ROS, reactive oxygen species; SCFAs, short-chain fatty acids; SFAs, saturated fatty acids; SGLT2, sodium-glucose cotransporter 2; SIRT1, sirtuin 1; SLD, steatotic liver disease; SOD, superoxide dismutase; SREBP-1c, sterol regulatory element-binding protein 1c; SREBP-2, sterol regulatory element-binding protein 2; T2DM, type 2 diabetes mellitus; T3, triiodothyronine; TAGs, triacylglycerols; TGF-β1, transforming growth factor-beta 1; TGR5, Takeda G protein-coupled receptor 5; THR-β, thyroid hormone receptor-beta; THs, thyroid hormones; TJs, tight junctions; TLR, toll-like receptor; TM6SF2, transmembrane 6 superfamily member 2; TNF-α, tumor necrosis factor-alpha; TSH, thyroid-stimulating hormone; UPFs, ultra-processed foods; VCTE, vibration-controlled transient elastography; VLDL, very low-density lipoprotein; WAT, white adipose tissue.
